# Arterial spin labeling performs comparably to 2‐[^18^F]fluoro‐2‐deoxy‐D‐glucose positron emission tomography for presurgical evaluation in pediatric lesional epilepsy

**DOI:** 10.1002/epi.70199

**Published:** 2026-03-14

**Authors:** Antonio Giulio Gennari, Dorottya Cserpan, Raimund Kottke, Niklaus Krayenbühl, Michael Messerli, Martin W. Hüllner, Ruth O'Gorman Tuura, Georgia Ramantani

**Affiliations:** ^1^ Department of Neuropediatrics University Children's Hospital Zurich Zurich Switzerland; ^2^ MR‐Research Center University Children's Hospital Zurich Zurich Switzerland; ^3^ Department of Nuclear Medicine University Hospital Zurich Zurich Switzerland; ^4^ Children's Research Center University Children's Hospital Zurich Zurich Switzerland; ^5^ Neuroscience Center Zurich, Eidgenössische Technische Hochschule Zurich Zurich Switzerland; ^6^ Department of Radiology University Children's Hospital Zurich Zurich Switzerland; ^7^ Division of Pediatric Neurosurgery University Children's Hospital Zurich Zurich Switzerland; ^8^ University of Zurich Zurich Switzerland

**Keywords:** epilepsy surgery, imaging, MRI, pediatric focal lesional epilepsy, PET

## Abstract

**Objective:**

This study was undertaken to test whether arterial spin labeling (ASL) performs comparably to 2‐[^18^F]fluoro‐2‐deoxy‐D‐glucose positron emission tomography (FDG‐PET), the mainstay functional imaging technique, in pediatric lesional epilepsy, while avoiding radiotracer exposure and additional sedation.

**Methods:**

We retrospectively included children with epilepsy due to focal cortical dysplasia, low‐grade epilepsy‐associated tumors, or hippocampal sclerosis who underwent standardized magnetic resonance imaging (MRI; including single‐delay ASL) and FDG‐PET during presurgical evaluation. Lesions, perilesional perfusion, and metabolic abnormalities were segmented and coregistered. Spatial overlap was quantified using DICE scores to compare functional modalities with each other (perfusion‐to‐metabolism: DICE_P‐to‐M_), with the lesion (metabolism‐to‐lesion: DICE_M‐to‐L_; perfusion‐to‐lesion: DICE_P‐to‐L_), and, in seizure‐free children, with the resection cavity (lesion‐, metabolism‐, perfusion‐to‐resection cavity: DICE_L‐/M‐/P‐to‐Post_). We also assessed the temporal stability of perilesional ASL abnormalities and the presence of remote ipsilateral/contralateral abnormalities. Equivalence testing used the Wilcoxon signed‐rank equivalence test with FDG‐PET as reference; Cohen *κ* quantified agreement for remote abnormalities.

**Results:**

Fifteen children were included; median ages at FDG‐PET and ASL were 7.7 and 7.5 years; 53% required sedation. Median perilesional volumes were 11 339 mm^3^ (FDG‐PET) and 10 791 mm^3^ (ASL); both were larger under sedation (*p* < .001). Perilesional volumes were equivalent (*p* = .037). Median DICE_M‐to‐L_ and DICE_P‐to‐L_ were .3 and .4; equivalence was confirmed (*p* < .001). Median DICE_P‐to‐M_ was .7, indicating strong ASL–FDG‐PET concordance. In seizure‐free children following surgery, DICE_M‐to‐Post_ and DICE_P‐to‐Post_ were both .6 and equivalent (*p* = .01). ASL findings were stable over time (DICE = .27–.75; *n* = 4 with repeat ASL). Remote ipsilateral abnormalities were common (ASL 73%, FDG‐PET 67%; *κ* = .53), with poor contralateral agreement (*κ* = .12).

**Significance:**

ASL yielded perilesional findings equivalent to FDG‐PET and showed comparable overlap with the resection cavity in seizure‐free children. As a radiation‐free technique embedded into routine MRI, ASL reduces logistics and avoids an additional sedation session. These findings support ASL as a practical alternative to FDG‐PET for presurgical workup, especially when FDG‐PET access is limited.

**Key points**

- ASL perilesional perfusion maps were equivalent to FDG‐PET metabolic maps (volume/DICE) and showed strong cross‐modal overlap.
- In seizure‐free children, ASL and FDG‐PET showed comparable overlap with the resection cavity, supporting surgical relevance.
- Sedation increased perilesional volumes for both modalities, but equivalence remained unchanged.
- Remote ipsilateral abnormalities were common, with moderate ASL–PET agreement; contralateral agreement was poor.
- ASL enables streamlined, radiation‐free presurgical workup and is a practical alternative when access to FDG‐PET is limited.

## 1 INTRODUCTION

Among children with epilepsy, ~ 25% develop drug resistance,^1^ with a brain lesion being a major determinant.^2^, ^3^ In carefully selected children with focal lesional epilepsy, surgery is safe and effective,^4^ offering curative potential—seizure freedom and antiseizure medication (ASM) discontinuation^5^—and improving long‐term neurodevelopment.^6^, ^7^, ^8^ Focal cortical dysplasia (FCD), low‐grade epilepsy‐associated tumors (LEATs), and hippocampal sclerosis (HS) are the most common underlying etiologies among surgical candidates.^9^, ^10^ Presurgical evaluation aims to localize the epileptogenic lesion, delineate the epileptogenic zone, and define eloquent regions.^5^ Magnetic resonance imaging (MRI) is crucial for guiding further exploration and expediting surgery.^11^, ^12^ Nonetheless, identifying a focal, resectable lesion on MRI—although prognostically favorable—does not guarantee seizure freedom.^13^ Advancing noninvasive techniques for surgical planning and outcome prognostication therefore remains essential.

Second‐line diagnostics can further localize the epileptogenic zone.^11^ Interictal 2‐[^18^F]fluoro‐2‐deoxy‐D‐glucose positron emission tomography (FDG‐PET) is the most widely used functional technique in pediatric epilepsy surgery^14^ and serves as a reference modality. FDG‐PET hypometabolism predicts postsurgical outcome,^15^ including in MRI‐negative cases.^16^ Arterial spin labeling (ASL)—an MRI‐based, noninvasive perfusion sequence—has also been linked to postsurgical seizure freedom in children with FCD‐ or LEAT‐associated focal epilepsy.^17^ Early studies suggest good agreement between FDG‐PET and ASL.^18^, ^19^, ^20^, ^21^, ^22^ However, most prior work focused on adults,^18^, ^20^, ^21^, ^23^, ^24^ mainly with temporal lobe epilepsy^21^, ^23^, ^24^ or without an MRI‐detectable lesion,^18^ used earlier generation ASL sequences,^18^, ^19^, ^20^, ^24^, ^25^ and relied primarily on visual analysis.^18^, ^23^ Testing equivalence, rather than simple correlation, is required to support the adoption of ASL for presurgical assessment and to inform postsurgical outcome prediction.

This study tested the equivalence between perilesional metabolic (FDG‐PET) and perfusion (ASL) abnormalities in pediatric focal epilepsy due to FCD, LEAT, or HS. Specifically, we (1) compared the extent of presurgical ASL hypoperfusion with FDG‐PET hypometabolism; (2) quantified similarity between modalities; (3) assessed the overlap of each modality with the epileptogenic lesion; and (4) in seizure‐free children following surgery, assessed overlap with the resection cavity.

## 2 MATERIALS AND METHODS

### 2.1 Patient selection

**FIGURE 1:**
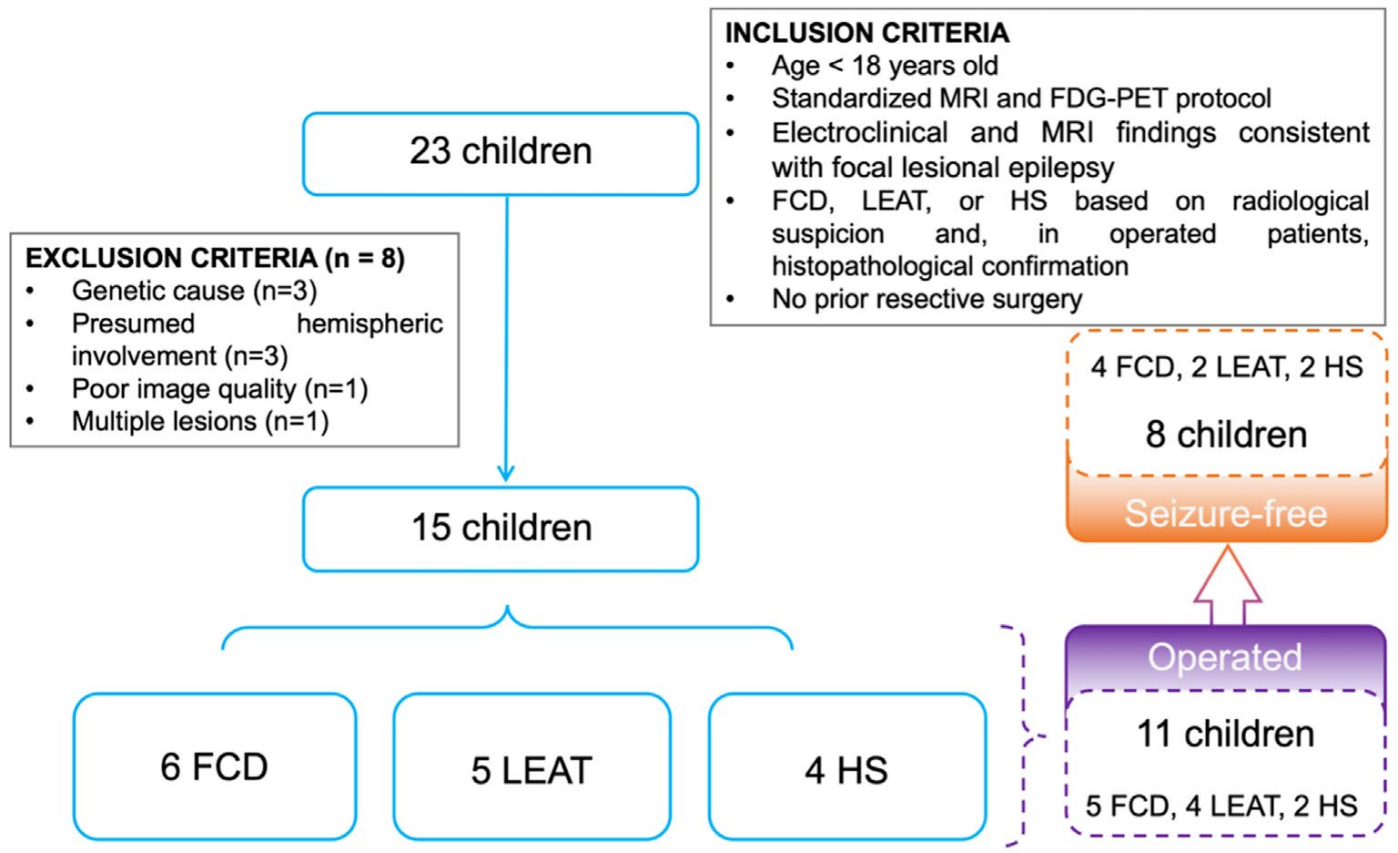
Patient selection flow chart. FCD, focal cortical dysplasia; FDG, 2‐[^18^F] fluoro‐2‐deoxy‐D‐glucose; HS, hippocampal sclerosis; LEAT, Low‐grade epilepsy‐associated tumor; MRI, magnetic resonance imaging; PET, positron emission tomography.

This retrospective, single‐center, cross‐sectional study included children with focal lesional epilepsy who underwent brain MRI at the University Children's Hospital Zurich and FDG‐PET at the University Hospital Zurich between July 2017 and August 2025 for presurgical evaluation. All patients were consecutive cases during the study period who met the following inclusion criteria: (1) age ≤ 18 years at the most recent scan; (2) MRI^26^, ^27^, ^28^ and FDG‐PET acquired according to standardized epilepsy protocols^29^; (3) electroclinical and MRI findings consistent with focal lesional epilepsy; (4) discrete epileptogenic lesions such as FCD, LEAT, or HS on MRI, confirmed by histopathology in operated patients; and (5) no prior resective surgery for tumors or epilepsy. Exclusion criteria were the following: (1) hemispheric epileptogenic lesions, (2) multiple lesions, (3) pathogenic epilepsy‐related genetic variants, and (4) poor‐quality MRI or FDG‐PET scans (Figure 1).

Clinical variables included age at epilepsy onset, epilepsy duration at the time of imaging, history of status epilepticus, and surgical features such as surgical procedure and postsurgical seizure outcome at last follow‐up in the operated subgroup. Seizure freedom was defined according to the Engel classification.^30^

Written informed consent for the use of clinical data was obtained from all parents and, where applicable, patients. The study was approved by the local ethics committee (Kantonale Ethikkommission Zürich, KEK‐ZH PB‐2024‐00298).

### 2.2 MRI and FDG‐PET acquisition

MRI scans were acquired on a 3‐T scanner (Discovery MR750 or Signa Premier; GE HealthCare) with an eight‐channel head coil, following the HARNESS (Harmonized Neuroimaging of Epilepsy Structural Sequences) protocol.^31^ The protocol was supplemented by a single‐delay ASL sequence with the following parameters: echo time = 10.5–11.2 ms, repetition time = 4531–4742 ms, postlabeling delay = 1525 ms, flip angle = 111°, three averages, slice thickness = 4 mm, slice spacing = 4 mm, and labeling duration = 1450 ms.^22^ The ASL sequence acquisition time was 4.68 min. ASL images were interpreted only within the standard radiology report and not used to guide treatment.^32^

FDG‐PET scans were performed on a PET/computed tomography (CT) system (20%; Discovery MI; GE HealthCare) or a PET/magnetic resonance (MR) system (80%; Signa; GE HealthCare), using structural images for attenuation correction and anatomical colocalization. Children fasted for ≥4 h before tracer injection.^22^ FDG dosage followed a weight‐based three‐tier protocol^29^ with a standardized uptake period of 50–60 min. All FDG‐PET scans were clinically interictal, and no seizures were observed during tracer uptake or image acquisition. Simultaneous electroencephalographic (EEG) monitoring was not performed. Sedation was administered if clinically indicated.^22^ In very young children or those with cognitive or behavioral difficulties, sedation was initiated before FDG injection to ensure that the child remained calm and still during the uptake phase, thereby avoiding agitation‐induced increases in cerebral metabolism that could compromise scan quality. Sedated children completed the uptake period in the scanner, whereas nonsedated children waited in a quiet environment with minimal stimulation. FDG‐PET visual interpretation was incorporated into the clinical presurgical evaluation.

### 2.3 Image postprocessing

#### 2.3.1 Lesion and perilesional perfusion and metabolic change segmentation

For lesion and perilesional analyses, we used the MRI scan acquired closest in time to the FDG‐PET. After NIfTI (Neuroimaging Informatics Technology Initiative) conversion, FCD and LEAT were manually segmented in 3D Slicer (https://www.slicer.org) by a board‐certified radiologist with 10 years of neuroradiology experience, creating regions of interest (ROIs) based on the three‐dimensional (3D) T1‐weighted volume, but also incorporating information from fluid‐attenuated inversion recovery and T2‐weighted images. In HS, cortical and subcortical structures, including hippocampal subfields, were segmented automatically^33^ using FreeSurfer (https://freesurfer.net). For hippocampal sclerosis, lesion volume was estimated by comparing the affected hippocampal volume with the contralateral hippocampus.

Perilesional perfusion (ASL) and metabolic (FDG‐PET) abnormalities were segmented when discrepancies between the lesional and contralateral hemispheres were visible in ≥2 slices.^34^ Perilesional abnormalities were defined as hypometabolic or hypoperfused regions adjacent to and spatially continuous with the lesion. Voxels belonging to the epileptogenic lesion were included in the perilesional abnormality masks, consistent with clinical FDG‐PET interpretation, in which functional abnormalities are assessed in relation to the structural lesion rather than restricted to voxels outside the MRI‐defined lesion. FDG‐PET segmentations were reviewed and, if needed, modified by a nuclear medicine physician, radiologist, and neuroradiologist with triple board certification and 17 years of experience, blinded to ASL and clinical data. All non‐FreeSurfer segmentations were smoothed using dilation‐erosion algorithms. In children with multiple MRIs, ASL‐defined perilesional abnormalities were compared for temporal stability.

Quantitative ASL and FDG‐PET analyses presented here were performed for research purposes only and did not influence surgical decisions.

#### 2.3.2 Resection cavity segmentation

In operated children, resection cavities were segmented on 3‐month postsurgical 3D T1‐weighted MRI using a semiautomated ROI expansion algorithm followed by manual correction and smoothing.^17^

#### 2.3.3 Segmentation comparison

**FIGURE 2:**
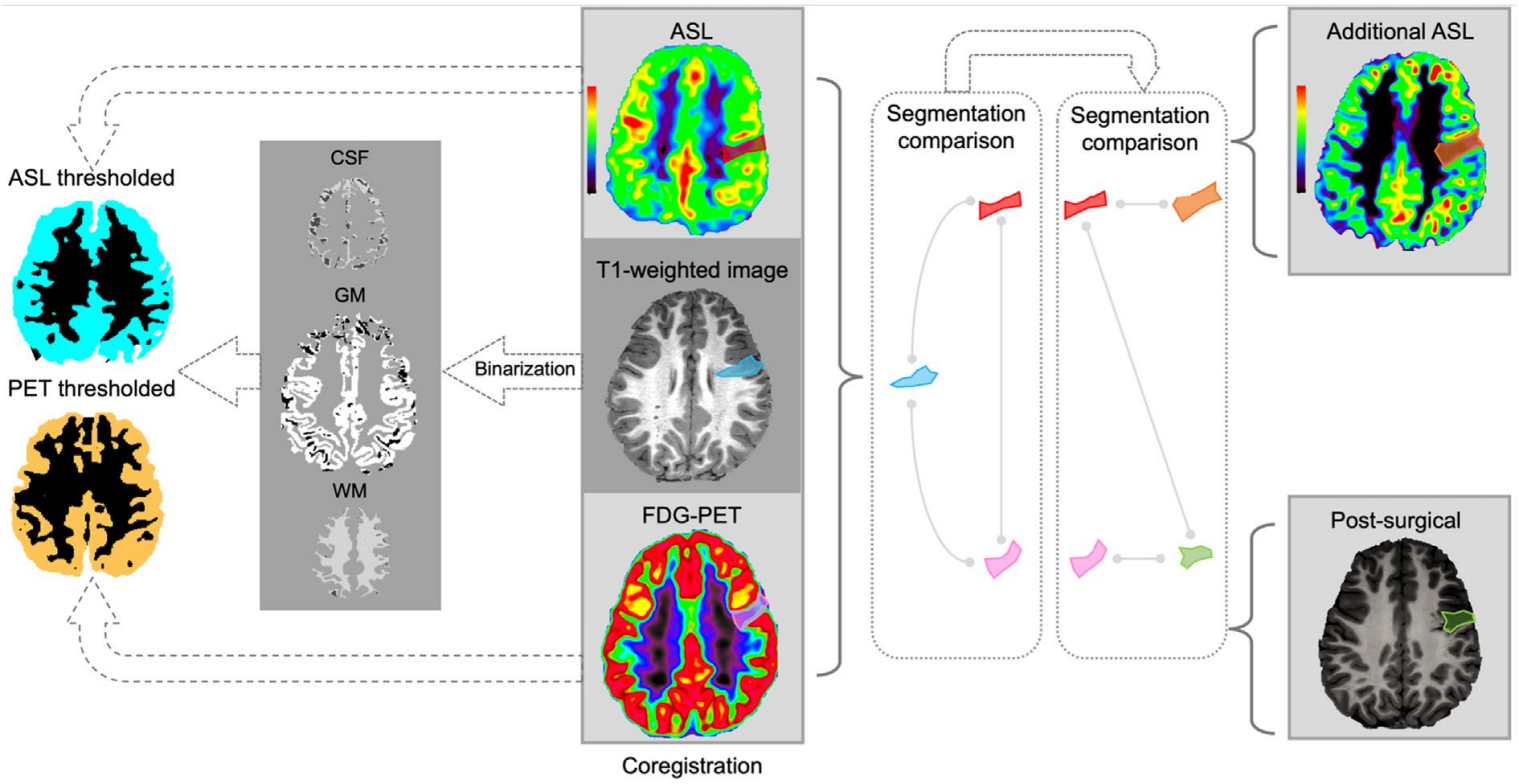
Image processing pipeline. Image preprocessing was performed using FSL, 3D Slicer, and FreeSurfer 7.3. T1‐weighted images were skull‐stripped and used for the segmentation of epileptogenic lesions. Perilesional perfusion (arterial spin labeling [ASL]) and metabolic (2‐[^18^F]fluoro‐2‐deoxy‐D‐glucose positron emission tomography [FDG‐PET]) abnormalities were manually segmented on ASL and FDG‐PET images, respectively. ASL and FDG‐PET images were coregistered to each subject's structural space using affine registration. Spatial overlap between perfusion and metabolic segmentations was quantified with the DICE score. To assess the stability of ASL findings, additional ASL scans (when available) were coregistered to the corresponding structural images and to the reference ASL scan, and perfusion change segmentations were compared. The surgical concordance of ASL‐ and FDG‐PET‐derived segmentations was evaluated by comparing preoperative segmentations with the resection cavity in patients who underwent resective surgery and achieved seizure freedom. To document remote findings, T1‐weighted images were segmented into cerebrospinal fluid (CSF), gray matter (GM), and white matter (WM) using FSL FAST. Mean GM perfusion and metabolism values were used to threshold ASL and FDG‐PET images. Remote perfusion or metabolic abnormalities were defined as hemispheric asymmetries located outside the perilesional masks and visible on at least two consecutive slices.

ASL and FDG‐PET scans were coregistered to skull‐stripped T1‐weighted images using FSL^18^, ^20^ (www.fmrib.ox.ac.uk/fsl). Pre‐ to postsurgical T1 scans were also registered in operated patients (Figure 2). Spatial overlap was quantified using the DICE score (fslr package in R; https://www.r‐project.org), calculated as twice the intersection divided by the sum of the two segmentations. Specifically, the metabolism‐to‐lesion and perfusion‐to‐lesion DICE scores (DICE_M‐to‐L_ and DICE_P‐to‐L_) measured overlap between hypometabolic or hypoperfused abnormalities and the epileptogenic lesion (ASL vs. structural lesion, FDG‐PET vs. structural lesion), whereas the perfusion‐to‐metabolism DICE score (DICE_P‐to‐M_) quantified the overlap between perfusion and metabolic abnormalities (ASL vs. FDG‐PET). The lesion‐, metabolism‐, or perfusion‐to‐resection cavity DICE scores (DICE_L‐to‐Post_, DICE_M‐to‐Post_, and DICE_P‐to‐Post_) assessed overlap between epileptogenic lesion, hypometabolic, or hypoperfused abnormalities and the resection cavity in seizure‐free children.

#### 2.3.4 Remote perfusion and metabolism analysis

Remote abnormalities outside the perilesional zone^22^ were evaluated ipsilaterally and contralaterally. Skull‐stripped 3D T1‐weighted images underwent bias‐field correction and gray matter, white matter, and cerebrospinal fluid segmentation using FSL Brain Extraction Tool (BET) and FMRIB’s Automated Segmentation Tool (FAST). Gray matter masks were used to calculate the mean hemispheric standardized uptake value (FDG‐PET) and mean gray matter cerebral blood flow (ASL), which served as lower thresholds for binarizing the functional maps. No additional cluster‐size filtering was applied. To reduce false positives, only abnormalities visible in at least two consecutive slices were considered. We avoided referencing mean gray matter standardized uptake value or cerebral blood flow to regions such as the cerebellum or basal ganglia due to common epilepsy‐related perfusion or metabolic abnormalities.^35^ Regions showing hemispheric discrepancies outside the perilesional masks and visible in ≥2 slices were classified as remote abnormalities. Remote abnormalities were defined as hemispheric perfusion or metabolic asymmetries outside the perilesional region, visible in at least two consecutive slices and assessed relative to the contralateral hemisphere to reduce physiological asymmetry.^34^ Because the functional deficit zone may extend well beyond the epileptogenic zone, segmentation was intentionally restricted to perilesional abnormalities to avoid misclassifying widespread functional deficit as remote changes.

### 2.4 Statistical analysis

All analyses were performed in R (version 4.0.5, https://www.r‐project.org). Normality was assessed with the Shapiro–Wilk test. Categorical variables were summarized as count and percentage, and continuous variables as median with interquartile range (IQR) or mean ± SD, depending on distribution. Comparisons between categorical variables were performed using chi‐squared or Fisher exact tests based on the counts. Continuous variables were compared using two‐tailed nonparametric tests (Kruskal–Wallis, Wilcoxon–Mann–Whitney). Correlations were analyzed with Spearman rank correlation. Statistical significance was set at *p* < .05, and Holm correction was applied for multiple comparisons.

Equivalence testing^36^ used the Wilcoxon signed‐rank equivalence test (TOSTER package) to compare segmented volumes of perilesional hypometabolic versus hypoperfused abnormalities, as well as their (1) overlap with the structural lesion (DICE_M‐to‐L_, DICE_P‐to‐L_) and (2) overlap with the resection cavity in seizure‐free patients (DICE_M‐to‐Post_, DICE_P‐to‐Post_). FDG‐PET served as the reference. The TOSTER package implements a two‐sided Wilcoxon signed‐rank test in addition to the equivalence testing. The results of both tests are reported. Equivalence bounds for volumetric analysis were set at ±2500 mm^3^ relative to FDG‐PET to account for modality differences, timing, and minimal coregistration error. This threshold was justified using proxy measures of intrinsic measurement error and segmentation variability derived from neuro‐oncology^37^ and epilepsy imaging studies.^27^, ^28^, ^38^, ^39^ The DICE equivalence threshold (±.20) was defined based on reported variability in pediatric brain tumor segmentation studies using structural MRI.^40^ Equivalence testing was chosen over a general inferential statistic framework because accepting the null hypothesis in a superiority test does not demonstrate similarity; equivalence directly tests whether differences lie within an acceptable margin.^41^

Agreement between FDG‐PET and ASL for remote ipsilateral and contralateral abnormalities was evaluated with Cohen *κ* and interpreted as poor (<.20), fair (≥.20 to <.40), moderate (≥.40 to <.60), good (≥.60 to <.80), and very good (≥.80 to 1). Ninety‐five percent confidence intervals (CIs) for *κ* were calculated using Wald's method.

## 3 RESULTS

### 3.1 Clinical features

Fifteen children were included in the study; eight (53%) were boys (Table 1). The median age at epilepsy onset was 3.6 years (IQR = 1–7). At the time of FDG‐PET and ASL, the median ages were 7.7 years (IQR = 5–10) and 7.5 years (IQR = 6–10), respectively. The median absolute interval between scans was 35 days (IQR = 20–56). The median administered FDG dose was 50 MBq (IQR = 36–59). Eight children (53%) required sedation for both FDG‐PET and MRI. Propofol was the primary sedative in all cases, with ketamine or nalbuphine used as adjuncts in 81%. Fourteen children (93%) remained on the same ASM regimen at both scans; the only exception, who had the longest interscan interval, had an increased dosage of two ASMs between MRI and FDG‐PET (Table 1 and Table S1). Eleven children (73%) underwent resective surgery, with histopathology confirming FCD in five (45%, four type IIb, one type IIa), LEAT in four (36%; three gangliogliomas), and HS in two (18%). All FCD and LEAT cases underwent lesionectomy; HS cases underwent amygdalohippocampectomy (Table S1).

**TABLE 1:** Patient characteristics (*N* = 15).

Characteristic		Value
Boys, *n* (%)		8 (53)
Lesion type, *n* (%)^a^	FCD	6 (40)
	LEAT	5 (33)
	HS	4 (27)
Age at epilepsy onset, years, median [IQR]		3.6 [1–7]
Epilepsy duration at the latest scan, years, median [IQR]		2.3 [1–4]
History of status epilepticus, *n* (%)		2 (13)
Lesion lateralization, right, *n* (%)		6 (40)
Lesion localization, *n* (%)	Frontal	6 (40)
	Temporal	7 (46)
	Occipital	1 (7)
	Multilobar^b^	1 (7)
Surgery performed, *n* (%)		11 (73)
Seizure freedom at the last follow‐up, *n* %		8 (80)
Lesion volume, mm^3^, median [IQR]		3208 [1211–8274]
Metabolic change volume, mm^3^, median [IQR]		11 339 [8149–20 815]
Perfusion change volume, mm^3^, median [IQR]		10 791 [5893–18 099]
Double ASL evaluations, *n* (%)		4 (27)

Abbreviations: ASL, arterial spin labeling; FCD, focal cortical dysplasia; HS, hippocampal sclerosis; IQR, interquartile range; LEAT, low‐grade epilepsy‐associated tumor.

^a^ As reported by pathology in operated cases and radiology in nonoperated ones.

^b^ FCD centered around the ascending ramus of the Sylvian fissure involving both the parietal and the temporal lobes.

Postoperative follow‐up was available for all but one patient (median = 9.6 months, IQR = 6.7–12.4). Eight of 11 children (72%) were seizure‐free at last follow‐up (Engel IA); three of them (38%) discontinued ASM. Of the remaining three operated patients, two had postoperative seizure recurrence due to incomplete lesion resection, whereas the remaining patient had no postoperative follow‐up at the time of analysis. Among the four children who did not undergo surgery, presurgical data were concordant in all cases; two were seizure‐free under medical treatment and therefore not offered surgery, and two had not yet undergone surgery at the time of data analysis.

### 3.2 Visual evaluation of lesional and perilesional findings

**FIGURE 3:**
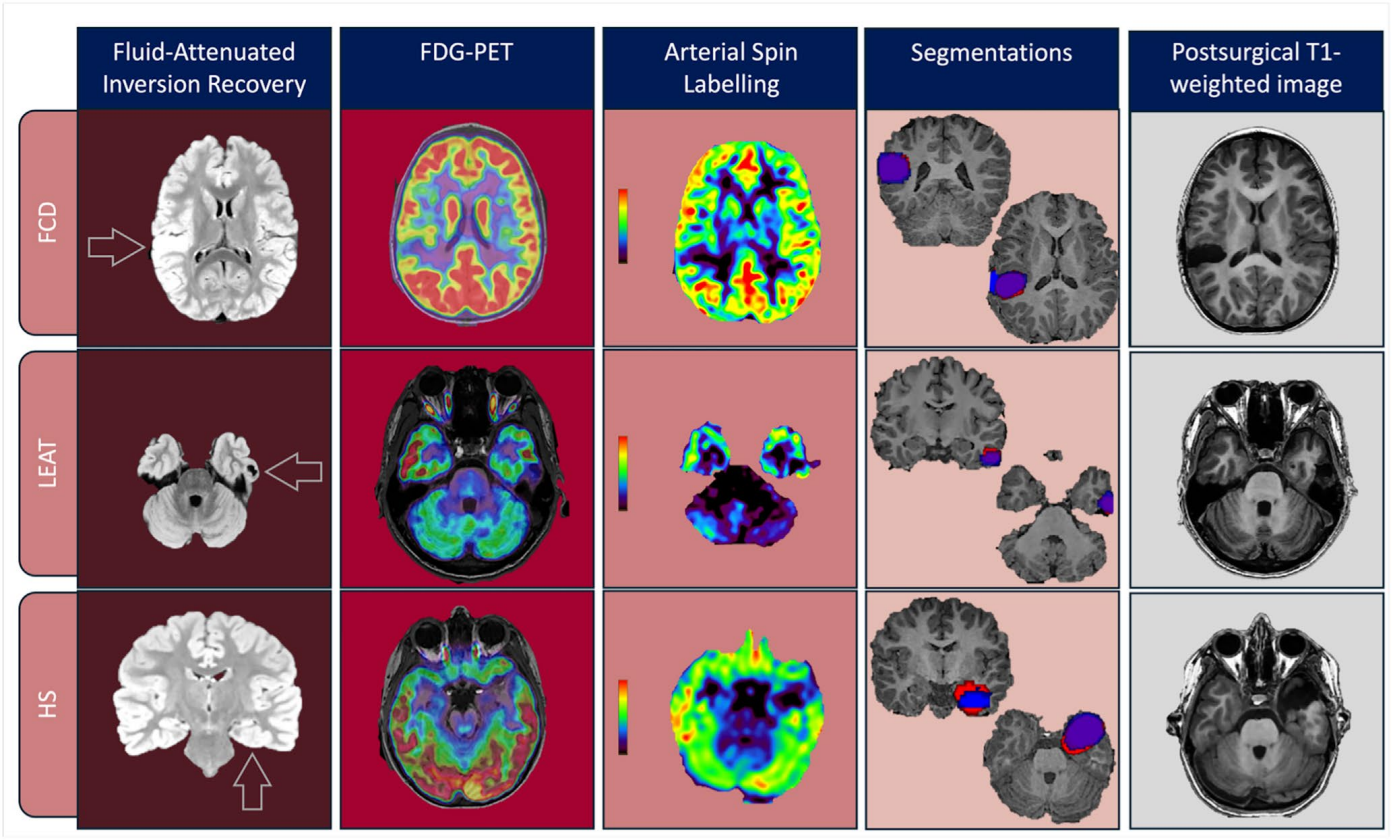
Illustrative cases. Three illustrative cases include a 4.2‐year‐old girl with multilobar right focal cortical dysplasia (FCD), a 16.3‐year‐old boy with left temporal ganglioglioma, and a 10.2‐year‐old girl with left hippocampal sclerosis (HS). Epileptogenic lesions (arrows) are shown on fluid‐attenuated inversion recovery images. 2‐[^18^F]Fluoro‐2‐deoxy‐D‐glucose positron emission tomography (FDG‐PET) and arterial spin labeling (ASL) revealed perilesional hypometabolism and hypoperfusion in all cases. Metabolic (red) and perfusion (blue) change segmentations, overlaid on T1‐weighted images, demonstrate strong agreement (DICE = .84, .63, and .72, respectively). Perfusion change segmentations (blue) are semitransparent to improve visualization. All patients underwent surgery and achieved seizure freedom (Engel IA). The concordance between FDG‐PET or ASL and resection cavities, as seen on postsurgical T1‐weighted images, was high across cases. LEAT, low‐grade epilepsy‐associated tumor.

Lesions involved the right hemisphere in eight children (53%). All but one lesion (93%) were unilobar: seven temporal, six frontal, and one occipital (Table 1). Hypoperfused and hypometabolic abnormalities colocalized with the structural lesion in all cases. All perilesional findings were hypometabolic on FDG‐PET and hypoperfused on ASL (Figure 3).

### 3.3 Quantitative findings

**FIGURE 4:**
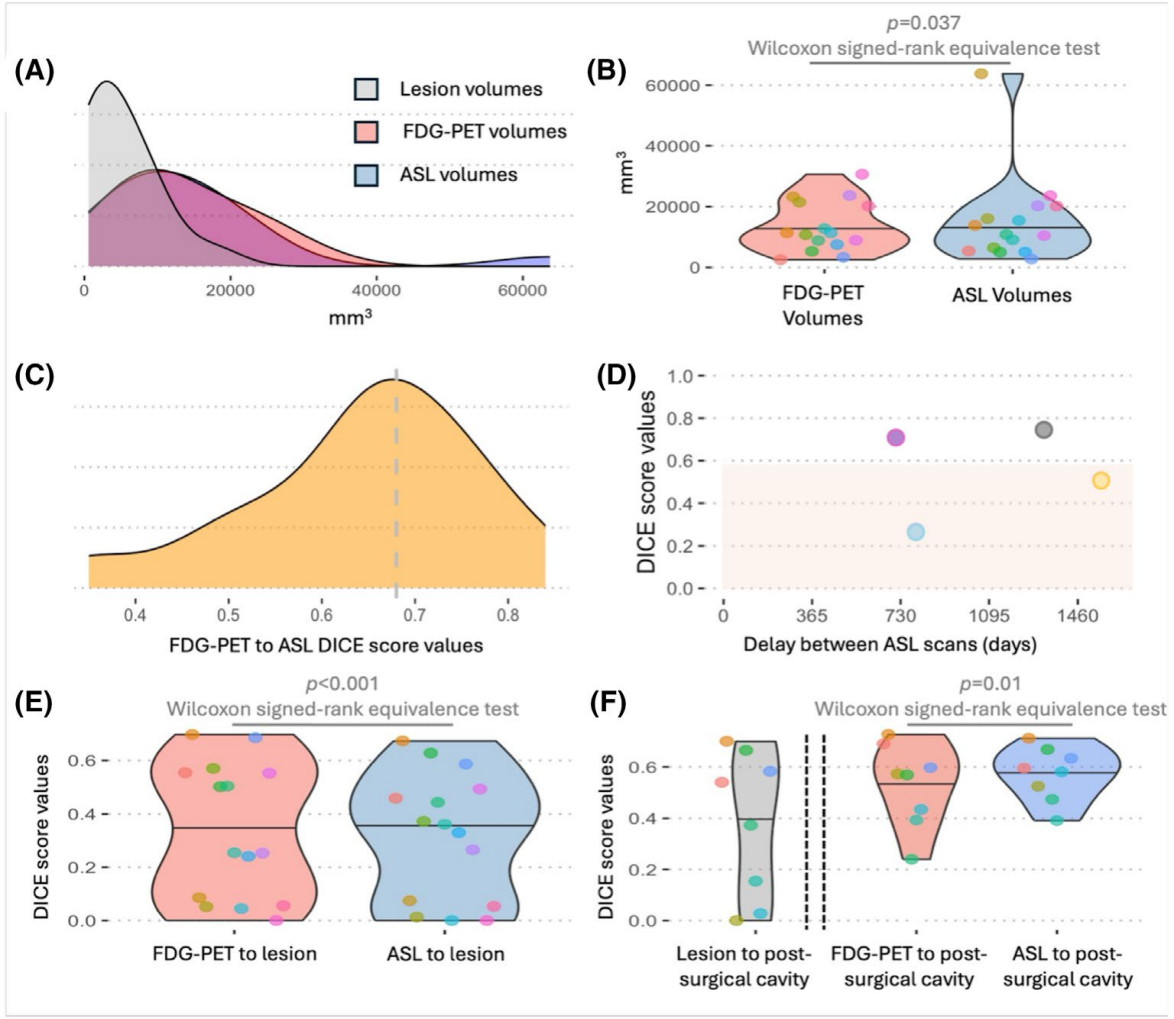
Descriptive metrics. (A) Density plots of lesion (gray), perilesional metabolic (2‐[^18^F]fluoro‐2‐deoxy‐D‐glucose positron emission tomography [FDG‐PET], red), and perfusion (arterial spin labeling [ASL], blue) volumes derived from structural MRI, 2‐[^18^F]fluoro‐2‐deoxy‐D‐glucose‐positron emission tomography (FDG‐PET), and arterial spin labeling (ASL). (B) Violin plots showing equivalence between perilesional FDG‐PET (red) and ASL (blue) segmentation volumes. (C) Density plot of FDG‐PET‐to‐ASL DICE scores; the dashed gray line marks the median. (D) Scatterplot showing similarity between ASL perilesional segmentations, expressed as DICE scores, as a function of the interval between scans, in patients who underwent repeat ASL imaging. Points are color‐coded for readability: yellow, purple, light blue, and light gray correspond to Cases 1, 4, 7, and 9 in Table S1, respectively. Cases highlighted by the transparent orange box had their first scan acquired before age 2 years. (E, F) Violin plots of DICE values showing overlap between FDG‐PET and ASL segmentations and the epileptogenic lesion, and between FDG‐PET and ASL segmentations and the resection cavity. Lesion values in panel F are shown for visualization only and were not used for equivalence testing. All violin plots include colored dots indicating patient‐level values.

Median lesion volume was 3208 mm^3^ (IQR = 1211–8274). HS cases had the smallest lesions (≤1000 mm^3^). Median perilesional change volumes were 11 339 mm^3^ (IQR = 8149–20 814) for FDG‐PET and 10 791 mm^3^ (IQR = 5893–18 099) for ASL (Figure 4A). Neither volume correlated with age at scan (*p =* .47 and *p =* .76, Spearman tests), epilepsy duration (*p =* .85 and *p =* .15, Spearman test), or age at epilepsy onset (*p =* .51 and *p =* .23, Spearman test), but both were significantly larger under sedation (*p* < .001, Mann–Whitney test).

### 3.4 Correlation between structural lesion, FDG‐PET, and ASL perilesional abnormalities

Median DICE_M‐to‐L_ and DICE_P‐to‐L_ scores were .3 (IQR = .1–.6) and .4 (IQR = .1–.5). Although overlap between lesion and perfusion abnormalities was minimally higher, equivalence was confirmed (Wilcoxon signed‐rank test, *V* = 78, *p* = .32; equivalence bound ±.2, *V* = 0, *p* < .001, Wilcoxon signed‐rank equivalence test; Figure 4E). Equivalence testing also confirmed that perilesional metabolic and perfusion abnormalities volumes were statistically equivalent (Wilcoxon signed‐rank test, *V* = 49, *p* = .551; equivalence bound ±2500 mm^3^, *V* = 22, *p* = .037, Wilcoxon signed‐rank equivalence test; Figure 4B). The median DICE_P‐to‐M_, reflecting spatial similarity between modalities, was .7 (IQR = .6–.7; Figure 4C).

### 3.5 Correlation between resection cavity, FDG‐PET, and ASL perilesional abnormalities in seizure‐free patients

Among the eight seizure‐free children with 3‐month postoperative MRI, median resection cavity volume was 9230 mm^3^ (IQR = 6356–12 116). Median DICE_L‐to‐Post_ was .5 (IQR = .1–.6), median DICE_M‐to‐Post_ was .6 (IQR = .4–.6), and DICE_P‐to‐Post_ was .6 (IQR = .5–.6). Equivalence between metabolic and perfusion overlaps with the resection cavity was confirmed (Wilcoxon signed‐rank test, *V* = 24, *p* = .441; equivalence bound ±.2, *V* = 1, *p* = .01, Wilcoxon signed‐rank equivalence test; Figure 4F).

### 3.6 Stability of ASL findings over time

Four children (two FCD, two HS) underwent two MRIs with ASL (Table S2 and Figure S1). Scan intervals were 709, 793, 1321, and 1557 days. Corresponding DICE scores were .27, .51, .71, and .75. Lower scores occurred in cases where the initial scan was performed before the age of 2 years (Figure 4D).

### 3.7 Correlation between FDG‐PET and ASL remote abnormalities

Hypometabolic and hypoperfused abnormalities remote from the perilesional zone but within the ipsilateral hemisphere were observed in 11 (73%) and 10 (67%) children, respectively. Among children with temporal lesions, ipsilateral abnormalities localized to the insula and frontal lobe^42^ in 57% (metabolism) and 50% (perfusion), respectively. Contralateral abnormalities occurred in nine (60%) and 11 (73%) children, respectively. None of the children who failed to achieve seizure freedom following surgery showed additional ipsilateral or contralateral hypometabolic abnormalities or ipsilateral hypoperfused abnormalities; one had contralateral hypoperfused abnormalities. Agreement between modalities was moderate for ipsilateral abnormalities (*κ* = .53, 95% CI = .1–1) and poor for contralateral abnormalities (*κ* = .12, 95% CI = 0–.6; Tables S3 and S4).

## 4 DISCUSSION

To our knowledge, this is the first study to quantify volumetric equivalence between FDG‐PET‐ and ASL‐derived findings in children with focal lesional epilepsy undergoing presurgical evaluation. Beyond volume, high DICE_P‐to‐M_ values indicated that both modalities mapped to the same locations. Equivalence of DICE scores for modality‐to‐lesion and modality‐to‐resection cavity comparisons further supports concordance. Although sedated children had more extensive perilesional abnormalities on both modalities, equivalence remained unchanged. Taken together, these results add to the evidence that ASL is a valuable tool for presurgical assessment in pediatric focal lesional epilepsy, directly linking perilesional perfusion and metabolic abnormalities.

ASL, FDG‐PET, and T1‐weighted MRI capture different aspects of brain imaging and are influenced by distinct physical and technical factors, including differences in spatial resolution, sensitivity, and susceptibility to artifacts. These factors can affect spatial correspondence, particularly at finer spatial scales. Accordingly, the observed overlaps should be interpreted as reflecting convergent patterns related to shared underlying pathological processes rather than direct equivalence between modalities. Modality‐specific differences, which were accounted for in the equivalence bounds, should be considered when translating these cross‐modal comparison results to clinical practice.

### 4.1 FDG‐PET and ASL agreement is unaffected by sedation

FDG‐PET hypometabolism and ASL hypoperfusion yielded equivalent perilesional results across FCD, LEAT, and HS. Perilesional metabolic and perfusion alterations volumes were larger in sedated children, but equivalence between modalities was preserved. This aligns with a previous ASL study^17^ indicating sedation‐insensitive functional results, despite the known antiseizure effects of propofol, which suppresses interictal epileptic discharges on the EEG of children undergoing MRI.^43^ Our volumetric equivalence extends previous reports^19^, ^20^ showing good FDG‐PET/ASL correlation. Interictal perfusion single photon emission computed tomography (SPECT) has been used to assess cerebral perfusion, but multiple studies have shown that it is less sensitive than FDG‐PET for localizing the functional deficit zone.^44^, ^45^ Its use in pediatric presurgical evaluation has decreased over the past 2 decades, largely because of radiation exposure, tracer logistics, and the need for an additional sedation session in young children. ASL avoids these limitations and can be acquired within the routine MRI protocol, offering a more practical perfusion‐based alternative at centers where SPECT is rarely performed.

Unlike atlas‐based^20^ or anatomofunctional parcellations^19^ used previously, we applied manual segmentations, which improve anatomical specificity but may reduce repeatability. Prior correlation studies also assessed overall agreement across positive and negative scans.^19^, ^20^ Voxelwise methods may improve precision,^18^ but subtle voxel differences rarely affect clinical decisions and complicate image postprocessing. By applying equivalence testing, we tested whether differences between FDG‐PET and ASL—potentially influenced by interictal fluctuations in the epileptogenic zone,^46^ a known confounder for functional modalities—remained within a prespecified equivalence margin. In the largest pediatric cohort scanned with both modalities, 20 of 45 (44%) were positive on both, and four of 45 (9%) showed incongruence.^22^ However, sparse phenotyping in that study limits comparison with our well‐characterized cohort, where all cases had positive perilesional findings on both modalities. A simultaneous FDG‐PET and ASL study in HS reported larger perfusion than metabolic abnormalities,^21^ but subjective analysis and lack of formal statistics limit comparability.

Although DICE_P‐to‐M_ can appear abstract, it jointly captures volume and location and is sensitive to minor segmentation disagreements. Our median DICE_P‐to‐M_ of .7 is within the range reported when benchmarking high‐performance artificial intelligence models for fetal brain structures^47^, ^48^ and pediatric brain tumors,^49^ supporting strong spatial agreement between FDG‐PET and ASL. Importantly, the variability reported in those studies (SD)^47^, ^48^, ^49^ is comparable to our equivalence bounds for DICE_M‐to‐L_ and DICE_P‐to‐L_, supporting the suitability of those margins.

### 4.2 Perilesional FDG‐PET and ASL abnormalities align with resection cavities in postsurgical seizure freedom

In seizure‐free children following surgery, perilesional hypometabolism (FDG‐PET) and hypoperfusion (ASL) showed moderate‐to‐high overlap with the resection cavity. Functional modality‐to‐resection DICE scores were slightly higher than lesion‐to‐resection overlap (median DICE_M‐to‐Post_/DICE_P‐to‐Post_ .6 vs. DICE_L‐to‐Post_ .5), quantitatively confirming the relevance of removing the entire epileptogenic lesion,^50^ and highlighting the added value of functional maps in presurgical planning. Because perilesional median metabolic and perfusion change volumes were roughly three times the structural lesion volume, many voxels deemed “normal” on lesion‐only maps were functionally abnormal. Although previous FDG‐PET^15^ and ASL studies^17^ and a meta‐analysis^16^ support that functional imaging is a valuable tool for delineating the epileptogenic network, our study was not designed to address whether complete resection of functional imaging abnormalities is required for seizure freedom. Previous literature has similarly shown that perilesional ASL abnormalities better predicted 1‐year postsurgical seizure freedom than lesion‐based assessment,^17^ and focal FDG‐PET hypometabolism predicted favorable long‐term outcomes independent of structural MRI.^16^ The stronger agreement between intraoperative electrocorticography (ECoG) and FDG‐PET than between ECoG and MRI^51^ further supports integrating functional imaging into presurgical evaluation.

### 4.3 Perilesional ASL‐derived perfusion abnormalities are stable over time

Across four children with repeat presurgical ASL scans (interval = 709–1557 days), perilesional hypoperfused abnormalities remained broadly consistent. Lower concordance occurred when the initial scan was acquired before age 2 years, reflecting developmental effects and the DICE score's sensitivity to minor boundary differences (Table S2 and Figure 1). The interpretation of repeated ASL scans may be further limited by developmental changes in brain perfusion over extended time intervals and by differences in sedation status between scans, both of which can affect perfusion measurements. These findings may suggest an influence of brain immaturity on ASL repeatability, although the sample size is too small to draw conclusions. Overall, these few cases illustrate that perilesional ASL abnormalities can persist over time, but larger cohorts are needed to assess reproducibility. All four children later underwent surgery and achieved seizure freedom, suggesting that early ASL may already capture the relevant perilesional network. Although preliminary, these findings support using ASL early in the evaluation of pediatric epilepsy, even before drug resistance is established, to image the perilesional epileptogenic network relevant for surgical planning.

### 4.4 Remote ASL‐derived perfusion abnormalities remain inconclusive

Remote FDG‐PET hypometabolic areas in the ipsilateral and contralateral hemispheres are well documented^52^ and occur in approximately one third of children with unilateral MRI lesions who undergo hemispherotomy,^35^ although their clinical relevance is unclear. Some studies suggest they may predict seizure recurrence,^53^ whereas others show limited association with postoperative seizure outcome^35^ and no impact on ambulatory status, spoken language skills, reading ability, or behavioral outcomes.^54^ In adults, ipsilateral metabolic abnormalities in temporal lobe epilepsy are common and reflect broader network involvement.^42^ In our cohort, all seizure‐free children following surgery had additional ipsilateral FDG‐PET hypometabolism, and 67% also had contralateral findings. In contrast, neither of the two non‐seizure‐free children had remote ipsilateral or contralateral abnormalities. Differences from prior reports likely reflect the small sample, differing etiologies, and methodological choices. Importantly, this is the first study to compare remote abnormalities across FDG‐PET and ASL, indicating that the two modalities do not provide interchangeable information in these regions.

### 4.5 Limitations and future directions

Limitations include small sample size, manual segmentation, and nonsimultaneous acquisition of ASL and FDG‐PET, introducing potential temporal variability. Furthermore, because FDG‐PET was not acquired simultaneously with EEG, subclinical ictal activity cannot be fully excluded, although no clinical seizures occurred during scanning. Moreover, the cohort included different underlying etiologies (FCD, LEATs, and HS), which may exhibit distinct perfusion and metabolic patterns, thereby limiting the generalizability of our findings. Additionally, because sedated and nonsedated children differed in age and lesion characteristics, the larger perfusion and metabolic abnormality volumes observed in the sedated subgroup cannot be attributed to sedation alone. The true effect of sedation cannot be determined without paired sedated and nonsedated scans in the same patients. In some cases, ASL and FDG‐PET were acquired several weeks or months apart, which may introduce interictal variability and affect cross‐modal comparisons. Nonetheless, this is the second‐largest pediatric cohort to date with both modalities^22^ and is well phenotyped. Expert evaluation remains the standard for comparing lesion detection across MRI sequences^55^ and functional modalities; even voxelwise studies applying asymmetry index or other advanced analyses^18^ ultimately relied on expert assessment. In our study, all segmentations were performed by experts with extensive experience in image analysis. Despite nonsimultaneous acquisitions, long‐interval repeat scans (~2 years) showed good concordance, supporting the stability of our findings. However, for the small subgroup with repeated ASL scans, differences in age, developmental stage, scan interval, and sedation status further limit the interpretability of these exploratory observations. Additionally, PET/CT and PET/MR systems were used across patients, introducing heterogeneity in image characteristics that could affect the segmentation of metabolic abnormalities and affect cross‐modal comparisons. Finally, because all children in our study had MRI‐positive lesions, our findings cannot be extrapolated to MRI‐negative or subtly lesional epilepsy, where FDG‐PET currently offers greater diagnostic value, and ASL requires further study.

Demonstrating equivalence between FDG‐PET and ASL enables clinical translation of existing FDG‐PET evidence to ASL, supporting broader use of this MRI‐based technique. Although larger studies are needed, our results suggest that ASL could serve as an alternative to FDG‐PET, benefiting resource‐limited settings and streamlining presurgical evaluation, potentially enabling expedited surgery.^56^ Moreover, because ASL is radiation‐free, it can be repeated when needed, particularly in older children who do not require sedation and in those with previously inconclusive findings on ASL or FDG‐PET.

## 5 CONCLUSIONS

ASL produced perilesional findings equivalent to FDG‐PET and aligned with resection cavities in seizure‐free children following surgery. As a radiation‐free technique acquired during the same MRI session, ASL can streamline presurgical evaluation and, in selected settings, serve as a substitute for FDG‐PET. Larger prospective studies should confirm these results and refine indications for ASL‐first pathways.

## AUTHOR CONTRIBUTIONS

Antonio Giulio Gennari, Georgia Ramantani, Ruth O'Gorman Tuura, Martin W. Hüllner, and Michael Messerli contributed to the study's conception and design. All authors contributed to data acquisition and analysis. Specifically, Antonio Giulio Gennari, Martin W. Hüllner, and Michael Messerli performed image analysis, and Dorottya Cserpan and Antonio Giulio Gennari carried out statistical analysis under the supervision of Georgia Ramantani. Antonio Giulio Gennari and Georgia Ramantani drafted the manuscript and prepared the figures, with significant contributions from Ruth O'Gorman Tuura and Dorottya Cserpan. Antonio Giulio Gennari, Dorottya Cserpan, Raimund Kottke, Niklaus Krayenbühl, Martin W. Hüllner, Michael Messerli, Ruth O'Gorman Tuura, and Georgia Ramantani reviewed, edited, and approved the final version of the manuscript.

## CONFLICT OF INTEREST STATEMENT

None of the authors has any conflict of interest to disclose.

## ETHICS STATEMENT

The collection and analysis of patient data were approved by and performed according to the guidelines and regulations of the local ethics committee (KEK‐ZH PB‐2024‐00298). We confirm that we have read the Journal's position on issues involved in ethical publication and affirm that this report is consistent with those guidelines.

## CONSENT

All parents gave written, informed general consent to reuse clinical data for research.

## Supporting information


Data S1.

## Data Availability

The data supporting this study's findings are available from the corresponding author upon reasonable request.
