# Supplementary material for: Arterial spin labeling performs comparably to 2‐[18F]fluoro‐2‐deoxy‐D‐glucose positron emission tomography for presurgical evaluation in pediatric lesional epilepsy
Source: Epilepsia. 2026 Mar 14;67(6):2940–53. doi: 10.1002/epi.70199 (PMC13285237; doi:10.1002/epi.70199)
Supplement: Supplementary file 1 — Data S1. [file EPI-67-2940-s001.docx]

**Supplementary Material**

[Supplementary Table 1 – Detailed patient characteristics 2](#_Toc216534455)

[Supplementary Table 2 – Detailed patient characteristics of patients who had repeated ASL scans 3](#_Toc216534456)

[Supplementary Table 3 – Distribution and Detectability of Additional Remote Metabolic and Perfusion Findings Ipsilateral to the Epileptogenic Lesion 4](#_Toc216534457)

[Supplementary Table 4 – Distribution and Detectability of Additional Remote Metabolic and Perfusion Findings Contralateral to the Epileptogenic Lesion 5](#_Toc216534458)

[Supplementary Figure 1 – Stability of ASL-derived perilesional findings over time 6](#_Toc216534459)

# **Supplementary Table 1 – Clinical, imaging, and surgical characteristics of the full cohort**

| **N.** | **Sex** | **Age at epilepsy onset, y** | **Epilepsy duration^$^, y** | **Status epilepticus** | **Seizure frequency** | **Age at MRI with ASL, y** | **Age at FDG-PET, y** | **FDG dose injected** | **ASM at scan** | **Sedation** | **Hemisphere** | **Lobar location** | **Surgery type** | **Lesion type** | **Post-surgical Outcome, Engel** |
| --- | --- | --- | --- | --- | --- | --- | --- | --- | --- | --- | --- | --- | --- | --- | --- |
| **1** | F | 1 | 4,8 | No | Weekly | 5,6 | 5,8 | 50 | LTG, OXC | Yes | Left | Frontal | Lesionectomy | FCD (IIB) | Ia |
| **2** | F | 0,9 | 3,3 | No | Daily | 4,2 | 4,2 | 50 | LTG, LEV, LCS | Yes* | Right | Multilobar^%^ | Lesionectomy | FCD (IIB) | Ia |
| **3** | M | 0,1 | 7,4 | No | Daily | 7,5 | 0,6^#^ | 44 | LTG, LEV^&^ | Yes* | Left | Frontal | Lesionectomy | FCD (IIA) | III |
| **4** | F | 8,3 | 1,9 | No | Monthly | 10,2 | 10,2 | 49 | LEV | No | Left | Temporal | Amygdalohippocampectomy | HS | Ia |
| **5** | M | 9,4 | 1,9 | No | Weekly | 11,3 | 11,3 | 84 | LEV | No | Right | Frontal | Lesionectomy | LEAT (GG) | IIIa |
| **6** | F | 3,6 | 2 | No | Weekly | 5,5 | 5,6 | 42 | STM, VPA | Yes* | Right | Frontal | Lesionectomy | FCD (IIB) | Ia |
| **7** | M | 0,3 | 2,2 | No | Daily | 2,4 | 2,5^#^ | 52 | OXC, LCS, CLB | Yes* | Right | Frontal | Lesionectomy | FCD (IIB) | Ia |
| **8** | F | 0,2 | 0,8 | No | Daily | 0,8 | 1 | 29 | LEV, CBZ | Yes* | Left | Temporal | Lesionectomy | LEAT (GG) | Ia |
| **9** | F | 6 | 3,8 | Yes | Daily | 9,6 | 9,8 | 66 | LEV | No | Left | Temporal | Amygdalohippocampectomy | HS | Ia |
| **10** | M | 11,6 | 1,1 | No | Weekly | 12,7 | 12,6 | 82 | OXC | No | Right | Frontal |  | LEAT | NA |
| **11** | M | 16,2 | 0,1 | No | Daily | 16,3 | 16,3 | 87 | BRV | No | Left | Temporal | Lesionectomy | LEAT(GG) | Ia |
| **12** | M | 4,9 | 1 | No | Monthly | 5,9 | 5,8 | 22 | OXC, CBZ | Yes* | Right | Temporal | Lesionectomy | LEAT | NA |
| **13** | M | 3,2 | 4,5 | No | Daily | 7,4 | 7,7^#^ | 50 | OXC | No | Left | Occipital | Not operated | FCD | NA |
| **14** | M | 5,2 | 2,7 | No | Weekly | 7,9 | 7,9 | 29 | OXC | No | Left | Temporal | Not operated | HS | NA |
| **15** | F | 3,2 | 4,5 | Yes | Daily | 7,6 | 7,7 | 29 | LTG | Yes | Left | Temporal | Not operated | HS | NA |

^#^: FDG-PET/CT acquisition; ^$^: epilepsy duration was calculated as the difference between the most recent scan (either ASL or FDG-PET) and age at epilepsy onset; ^@^: Lesion type based on radiological suspicion in the absence of histopathological confirmation; ^%^: Lesion centered around the ascending ramus of the Silvian fissure involving both parietal and temporal lobes; * Patients sedated for both MRI and FDG-PET acquisitions, using different sedation regimens; ^&^: Anti-seizure medication (ASM) protocol differed between FDG-PET and ASL scans; the ASM listed corresponds to the ASL scan; ASL: arterial spin labelling; ASM: anti-seizure medication; BRV: brivaracetam; CBZ: carbamazepin; CLB: clobazam; CT: computed tomography; FCD: focal cortical dysplasia; FDG: 2-[^18^F] fluoro-2-deoxy-D-glucose; GG: ganglioglioma; HS: hippocampal sclerosis; LCS: lacosamide; LEAT: low-grade epilepsy-associated tumor; LEV: levetiracetam; LTG: lamotrigine; OXC: oxcarbazepine; PET: positron emission tomography; STM: sulthiame; VPA: valproic acid.

# **Supplementary Table 2 – Clinical and imaging characteristics of patients with repeated ASL scans**

| **N.** | **Sex** | **Age at epilepsy onset, y** | **Epilepsy duration, y** | **Status epilepticus** | **Seizure frequency** | **Age at ASL, y** | **ASM at scan** | **Sedation** | **Hemisphere** | **Lobar location** | **Surgery type** | **Lesion type** | **Findings on structural images** |
| --- | --- | --- | --- | --- | --- | --- | --- | --- | --- | --- | --- | --- | --- |
| **1^@^** | F | 1 | 4,6 | No | Weekly | 5,6 | LTG, OXC | Yes | Left | Frontal | Lesionectomy | FCD (IIB) | **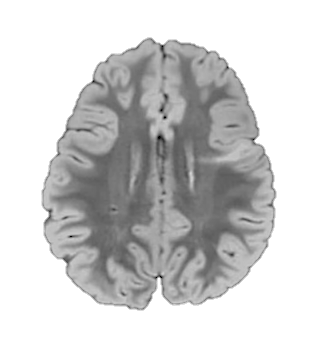** |
|  |  |  | 1,4 |  | Daily | 2,4 | None | Yes |  |  |  |  |  |
| **4** | F | 8,3 | 1,9 | No | Monthly | 10,2 | LEV | No | Left | Temporal | Amygdalohippocampectomy | HS | **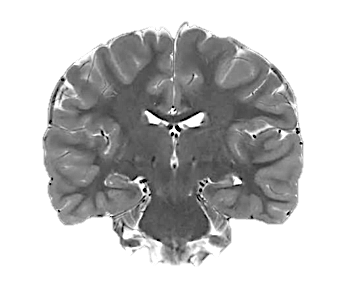** |
|  |  |  | 0,1 |  | First episode | 8,2 | None | No |  |  |  |  |  |
| **7** | M | 0,3 | 2,1 | No | Daily | 2,4 | OXC, LCS, CLB | Yes | Right | Frontal | Lesionectomy | FCD (IIB) | **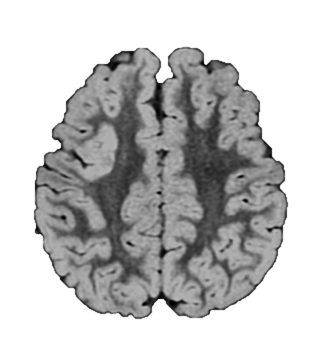** |
|  |  |  | 0 |  | Daily | 0,3 | None | Yes |  |  |  |  |  |
| **9^$^** | F | 6 | 3,6 | Yes | Daily | 9,6 | LEV | No | Left | Temporal | Amygdalohippocampectomy | HS | **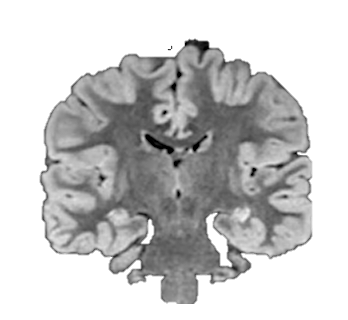** |
|  |  |  | 0 |  | First episode | 6 | LEV, DZP, MDZ* | Yes |  |  |  |  |  |

For each patient, characteristics are reported separately for each ASL acquisition. ^@^: Case B in **Supplementary Figure 1**; ^$^: Case A in **Supplementary Figure 1**; *: ASL scan acquired during admission for status epilepticus under diazepam and midazolam sedation; no further clinical seizures occurred during hospitalization; ASL: arterial spin labelling; ASM: anti-seizure medication; CLB: clobazam; DZP: diazepam; FCD: focal cortical dysplasia; MDZ: midazolam; HS: hippocampal sclerosis; LCS: lacosamide; LEV: levetiracetam; LTG: lamotrigine; OXC: oxcarbazepine

# **Supplementary Table 3 – Distribution and Detectability of Additional Remote Metabolic and Perfusion Findings Ipsilateral to the Epileptogenic Lesion**

|  |  | Ipsilateral Remote Perfusion Changes | | | | | | | | | | | | | | | |
| --- | --- | --- | --- | --- | --- | --- | --- | --- | --- | --- | --- | --- | --- | --- | --- | --- | --- |
| **Additional ipsilateral hypometabolic areas** |  | SFG | MFG | IFG | Cing | PCG | PoCG | SPL | IPL | Insula | STG | MTG | ITG | Lin & Fus | MOG | Cun | Not present |
|  | SFG | **2** |  |  |  |  |  |  |  |  |  |  |  |  |  |  | **1** |
|  | MFG |  |  |  |  |  |  |  |  |  |  |  |  |  |  |  |  |
|  | IFG |  |  | **1** |  |  |  |  |  |  |  |  |  |  |  |  | **2** |
|  | Cing |  |  |  |  |  |  |  |  |  |  |  |  |  |  |  | **1** |
|  | PCG |  |  |  |  |  |  |  |  |  |  |  |  |  |  |  |  |
|  | PoCG |  |  |  |  |  | **1** |  |  |  |  |  |  |  |  |  |  |
|  | SPL |  |  |  |  |  |  | **1** |  |  |  |  |  |  |  |  |  |
|  | IPL |  |  |  |  |  |  |  | **2** |  |  |  |  |  |  |  |  |
|  | Insula |  |  |  |  |  |  |  |  |  |  |  |  |  |  |  | **2** |
|  | STG |  |  |  |  |  |  |  |  |  | **1** |  |  |  |  |  |  |
|  | MTG |  |  |  |  |  |  |  |  |  |  | **1** |  |  |  |  | **3** |
|  | ITG |  |  |  |  |  |  |  |  |  |  |  | **1** |  |  |  |  |
|  | Lin & Fus |  |  |  |  |  |  |  |  |  |  |  |  | **1** |  |  |  |
|  | MOG |  |  |  |  |  |  |  |  |  |  |  |  |  | **1** |  |  |
|  | Cun |  |  |  |  |  |  |  |  |  |  |  |  |  |  |  |  |
|  | Not present | **1** | **1** | **1** |  |  | **1** |  |  | **1** |  | **1** |  |  | **1** |  |  |

SFG: superior frontal gyrus; MFG: middle frontal gyrus; IFG: inferior frontal gyrus, including the opercular region; Cing: Cingulate gyrus; PCG: pre-central gyrus; PoCG: post-central gyrus; SPL: superior parietal lobule; IPL: inferior parietal lobule; STG: superior temporal gyrus; MTG: middle temporal gyrus; ITG: inferior temporal gyrus; Lin & Fus: lingular and fusiform gyri, including temporo-mesial structures; MOC: middle occipital gyrus; Cun: cuneus

# **Supplementary Table 4 – Distribution and Detectability of Additional Remote Metabolic and Perfusion Findings Contralateral to the Epileptogenic Lesion**

|  |  | Contralateral Remote Perfusion Changes | | | | | | | | | | | | | | | |
| --- | --- | --- | --- | --- | --- | --- | --- | --- | --- | --- | --- | --- | --- | --- | --- | --- | --- |
| **Additional contralateral hypometabolic areas** |  | SFG | MFG | IFG | Cing | PCG | PoCG | SPL | IPL | Insula | STG | MTG | ITG | Lin & Fus | MOG | Cun | Not present |
|  | SFG |  |  |  |  |  |  |  |  |  |  |  |  |  |  |  |  |
|  | MFG |  | **1** |  |  |  |  |  |  |  |  |  |  |  |  |  |  |
|  | IFG |  |  |  |  |  |  |  |  |  |  |  |  |  |  |  |  |
|  | Cing |  |  |  |  |  |  |  |  |  |  |  |  |  |  |  |  |
|  | PCG |  |  |  |  | **1** |  |  |  |  |  |  |  |  |  |  |  |
|  | PoCG |  |  |  |  |  | **1** |  |  |  |  |  |  |  |  |  | **1** |
|  | SPL |  |  |  |  |  |  |  |  |  |  |  |  |  |  |  | **1** |
|  | IPL |  |  |  |  |  |  |  |  |  |  |  |  |  |  |  | **2** |
|  | Insula |  |  |  |  |  |  |  |  | **1** |  |  |  |  |  |  |  |
|  | STG |  |  |  |  |  |  |  |  |  |  |  |  |  |  |  |  |
|  | MTG |  |  |  |  |  |  |  |  |  |  | **2** |  |  |  |  | **3** |
|  | ITG |  |  |  |  |  |  |  |  |  |  |  | **1** |  |  |  |  |
|  | Lin & Fus |  |  |  |  |  |  |  |  |  |  |  |  |  |  |  | **1** |
|  | MOG |  |  |  |  |  |  |  |  |  |  |  |  |  |  |  | **2** |
|  | Cun |  |  |  |  |  |  |  |  |  |  |  |  |  |  |  |  |
|  | Not present |  | **2** | **3** |  |  | **1** | **1** |  |  | **1** |  |  | **1** | **2** | **2** |  |

SFG: superior frontal gyrus; MFG: middle frontal gyrus; IFG: inferior frontal gyrus, including the opercular region; Cing: Cingulate gyrus; PCG: pre-central gyrus; PoCG: post-central gyrus; SPL: superior parietal lobule; IPL: inferior parietal lobule; STG: superior temporal gyrus; MTG: middle temporal gyrus; ITG: inferior temporal gyrus; Lin & Fus: lingular and fusiform gyri, including temporo-mesial structures; MOC: middle occipital gyrus; Cun: cuneus

# **Supplementary Figure 1 – Stability of ASL-derived perilesional findings over time**

**
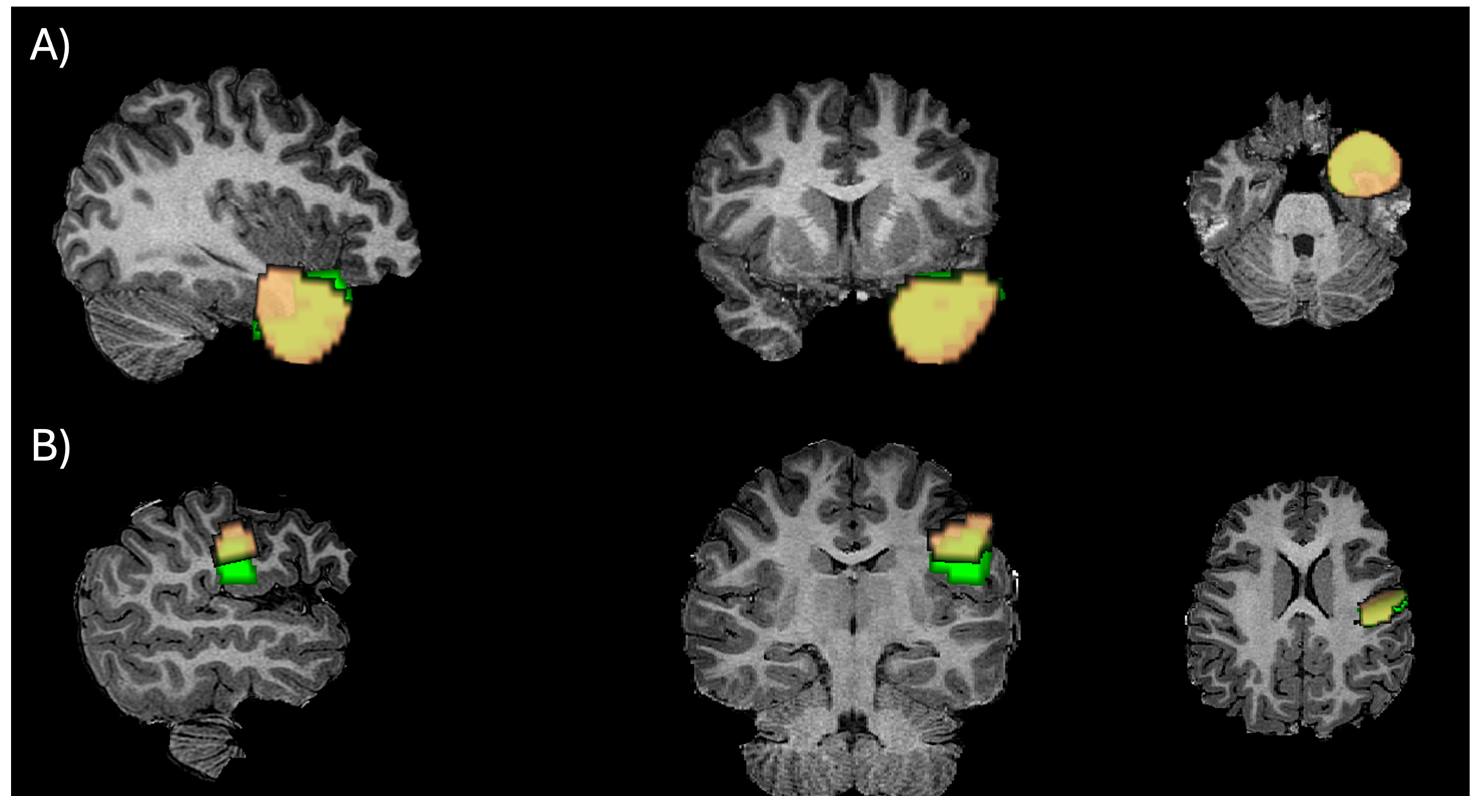
**

Two illustrative cases of focal lesional epilepsy are shown: (A) hippocampal sclerosis (HS) and (B) focal cortical dysplasia (FCD; same as Fig. 2), each with multiple ASL scans obtained 1’321 and 1’557 days apart, respectively. Green segmentations indicate the most recent scan, while orange segmentations indicate the preceding scan. Both patients underwent resective surgery and achieved seizure freedom. The scans show similar segmentation patterns over time; however, case A demonstrated a higher DICE score (0.75) than case B (0.51), illustrating how minor boundary differences can substantially affect this metric.

ASL: arterial spin labelling; FCD: focal cortical dysplasia; HS: hippocampal sclerosis
